# Acute renal failure as a form of presentation of sarcoidosis in a young adult: a case report

**DOI:** 10.1186/1752-1947-8-274

**Published:** 2014-08-13

**Authors:** Joana Rema, Margarida Carvalho, Raquel Vaz, Margarida Fonseca, Susana Sampaio, Augusta Praça, Margarida Freitas-Silva, Teresa Cardoso

**Affiliations:** 1 Department of Internal Medicine, Centro Hospitalar de São João, E.P.E, Alameda Professor Hernâni Monteiro, 4200-319 Porto, Portugal; 2 Department of Nephrology, Centro Hospitalar de São João, E.P.E, Alameda Professor Hernâni Monteiro, 4200-319 Porto, Portugal

**Keywords:** Acute renal failure, Granulomatous tubulointerstitial nephritis, Sarcoidosis

## Abstract

**Introduction:**

Sarcoidosis is a systemic granulomatous disease. Renal involvement is a rare initial presentation of this disease. Few articles on renal involvement as an initial presentation of sarcoidosis have been published in the literature.

**Case presentation:**

A 26-year-old Caucasian woman presented with acute renal failure as an initial manifestation of sarcoidosis.

**Conclusions:**

Renal involvement is an uncommon feature of sarcoidosis and it is essential to establish a fast and correct diagnosis because early therapy avoids progression to terminal renal failure.

## Introduction

Sarcoidosis is a systemic disease caused by noncaseating granulomas in multiple organs [1]. Its etiology is unknown [2]. In Caucasians, the prevalence is estimated to be 20 to 50 per 100,000 people [3]. The incidence of the disease is 16.5/100,000 and 19/100,000 in men and women, respectively [4]. Renal manifestations of sarcoidosis include changes in calcium metabolism, nephrocalcinosis and nephrolithiasis. Some studies have shown that granulomatous tubulointerstitial nephritis is associated with acute renal failure and may lead to hemodialysis. The standard treatment is based on corticosteroids. Failure to respond to corticosteroids or relapse after withdrawal may be associated with worsening of renal failure, requiring dialysis and even kidney transplantation [5].

## Case presentation

**Figure 1:**
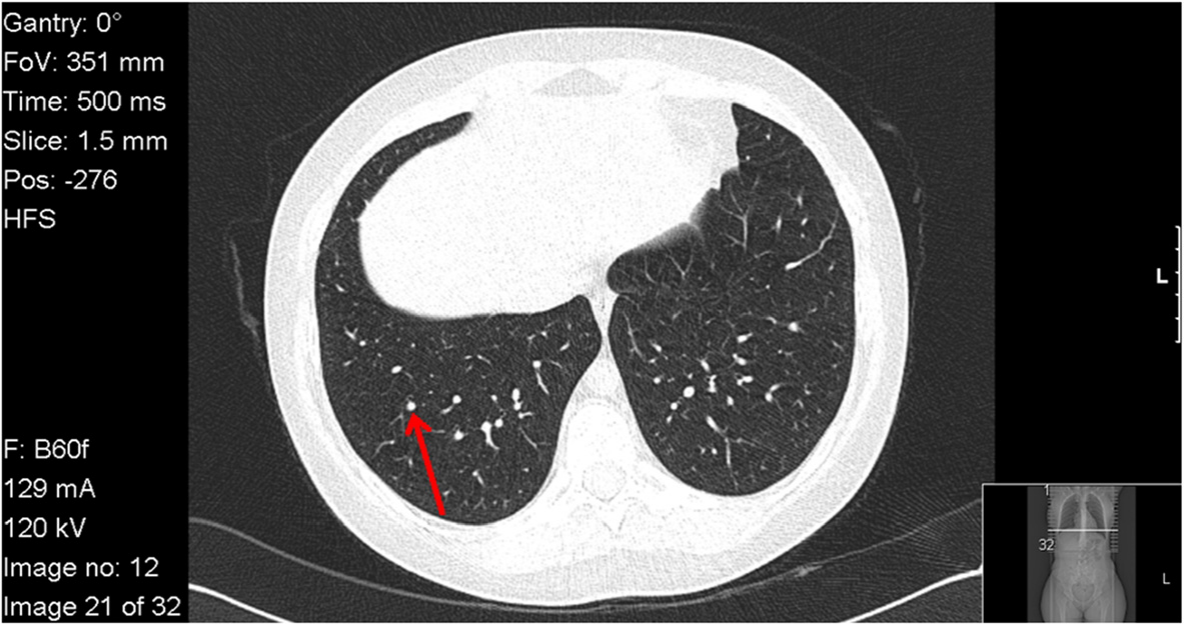
Thorax computed tomography scan of the patient, showing micronodules (indicated by an arrow).

**Figure 2:**
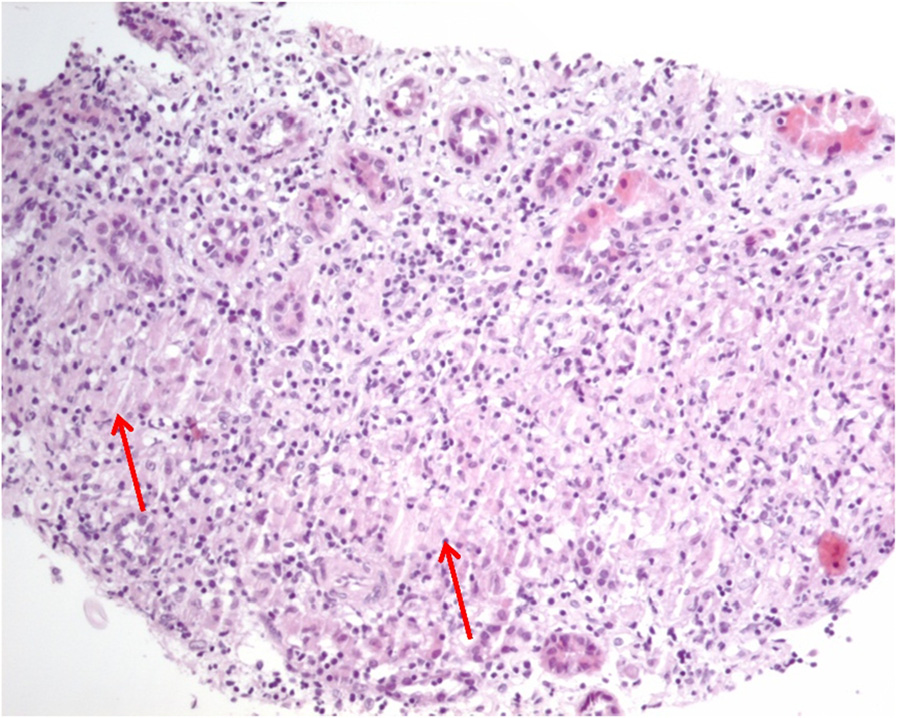
Renal biopsy of the patient showing interstitial inflammatory infiltrate with noncaseating granulomas (indicated by arrows) compatible with the diagnosis of sarcoidosis.

A 26-year-old Caucasian woman with a previous history of nephrolithiasis with renal colic episodes for the last 8 years, presented in the emergency department with nausea and anorexia. Her laboratory blood count revealed anemia with hemoglobin of 11.5g/dL (normal range: 12 to 16), leucopenia 3.61×10^9^/L (4 to 11), acute renal failure with blood urea nitrogen of 43mg/dL (10 to 50) and serum creatinine 1.6mg/dL (0.6 to 1.1). The urinary sediment had 0.3g/L proteins and erythrocytes (most did not lyse). There were no eosinophils. The objective examination remained with no alterations. No fever was documented. On analysis, we observed that her renal function was worsening with maximum creatinine of 2mg/dL on the 3rd day. The inflammatory markers in the form of C-reactive protein were 45mg/L (<3.0mg/L). There was hypercalcemia of 2.88mEq/L (2.26 to 2.64) with correction by albuminemia giving ionized calcium of 5.9mg/dL (4.6 to 5.4mg/dL). Her angiotensin-converting enzyme was elevated: 76U/L (<52U/L). Her 24-hour urine revealed no hypercalciuria and the assay of total protein per 24 hours was 1.03g. Her thyroid function was unchanged. Her parathormone was normal. An immunological study was negative. Viral serology showed no evidence of an acute infection; mycobacteriologic infection was excluded. A chest X-ray revealed a bilateral hilar lymphadenopathy. Abdominal and renal ultrasound documented splenomegaly, enlarged kidneys (right, 13.5cm; left, 13.9cm) with regular contours and preservation of the sinus parenchyma differentiation. A chest computed tomography revealed pulmonary micronodules (Figure 1). Pulmonary function tests revealed no alterations.A renal biopsy was performed and revealed nine glomeruli in light microscopy, one sclerotic, and the presence of tubular necrosis and tubular atrophy. The renal biopsy showed interstitial severe inflammatory infiltrate lymphocytes and plasma cells, eosinophils and epithelioid macrophages with formation of granulomas with giant cells (Figure 2).

The histopathology report indicated granulomatous tubulointerstitial nephritis compatible with the diagnosis of sarcoidosis.

**Figure 3:**
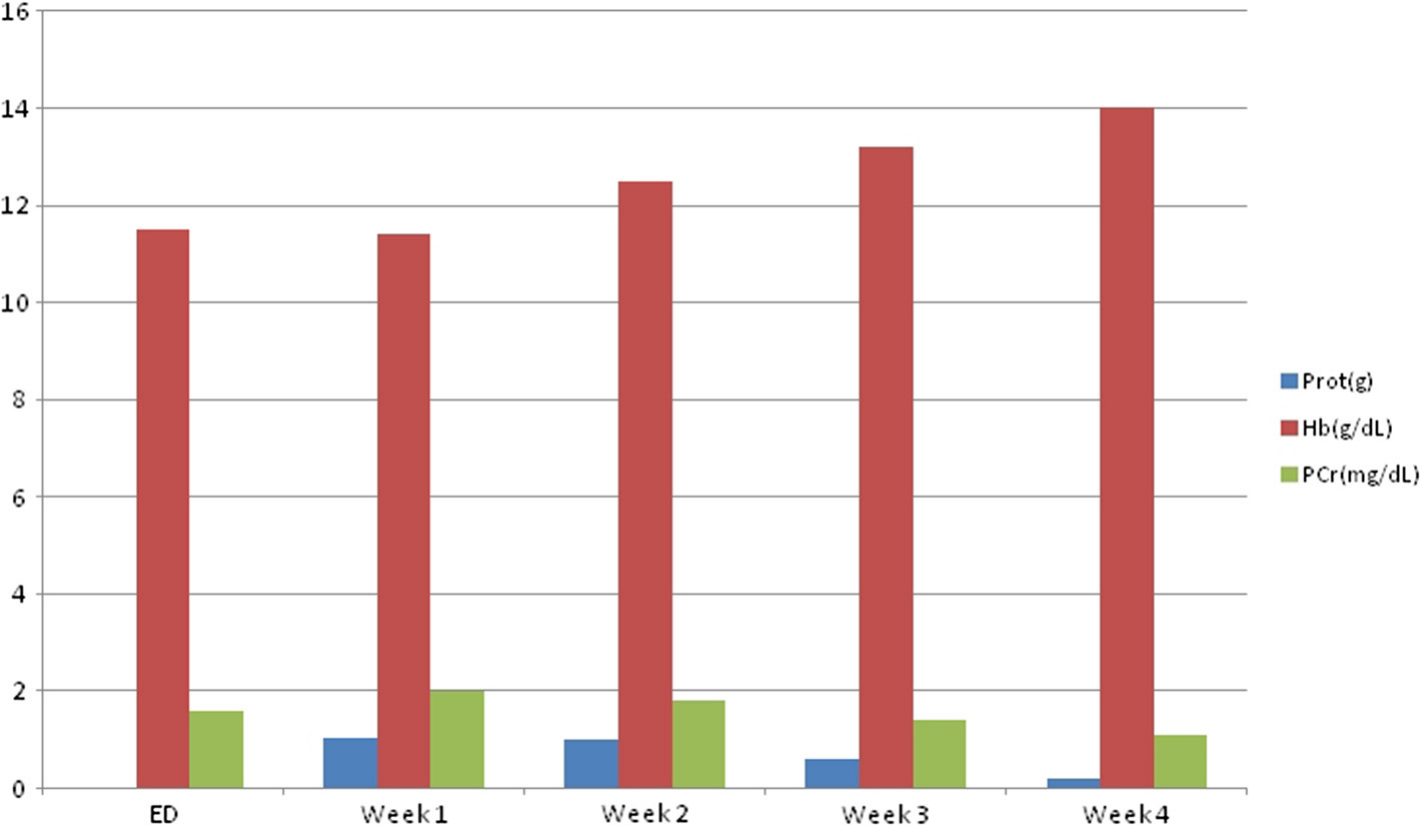
**Graphic showing the evolution of proteinuria per 24 hours; hemoglobin and renal function from admission to our emergency department to week 4.** Corticosteroids were started at the end of the first week. Abbreviations: ED, emergency department; Hb, hemoglobin; PCr, plasma creatinine; Prot, proteinuria per 24 hours.

Other systems were studied for the possible involvement of sarcoidosis and revealed no alterations: the patient was submitted to an eye examination to exclude uveitis, there were no skin or articular lesions such as erythema nodosum, lupus pernio or arthritis, no liver function test abnormalities, an electrocardiogram revealed no intraventricular conduction defect or nodal block and an echocardiogram was also normal. She had no neurological signs namely cranial neuropathy.She started corticosteroid initially with three pulses of methylprednisolone 1g daily for 3 consecutive days and then steroids in the form of oral prednisolone 1mg/kg/day. On further observation after 3 weeks of steroid therapy, she presented with no anemia or leucopenia, and a normalization of her renal function and proteinuria per 24 hours (Figure 3) was observed.

In terms of follow-up, she has been followed under an out-patient regimen; her hemogram and renal function remain unchanged and are associated with no alterations in physical examination despite corticosteroid weaning.

## Discussion

Sarcoidosis is a multisystem disease of unknown etiology; the lungs and lymph nodes are most commonly involved (30 to 60% of cases). Hypercalcemia (2 to 10%) and hypercalciuria (6 to 30%) can cause nephrocalcinosis and nephrolithiasis. The prevalence of tubulointerstitial nephritis varies between 7 and 27%, and spite of chronic renal failure develops in less than 1% of cases according to different retrospective studies [6]. The spectrum of renal involvement in sarcoidosis includes hypercalcemia, nephrocalcinosis and interstitial granulomas [7]. Glomerular involvement in sarcoidosis is not common, although focal segmental sclerosis, membranous glomerulonephritis, mesangioproliferative glomerulonephritis, mesangiocapillary glomerulonephritis, immunoglobulin A nephropathy, and crescentic glomerulonephritis have all been described although their mechanisms are not known. Membranous nephropathy has been most commonly reported. Enhanced production of immunoglobulins may be involved in the pathogenesis of membranous glomerulonephritis, but neither an antigen nor a specific antibody has been identified [8].

Sarcoidosis is not only a pulmonary or renal disease but a systemic disease that can affect any organ in the body. The most common extrapulmonary organs affected are the eye and skin. Other than the lungs, neurological and cardiac involvement must be excluded. The heart can manifest with mechanical and/or conduction defects. Central nervous system manifestations may present with cranial nerve symptoms (for example, VII, IX, X), with signs of optic chiasm involvement (for example, papilledema, blurred vision), meningeal symptoms caused by aseptic meningitis, symptoms of stroke/transient ischemic attack, and paresthesias. Patients may present with ophthalmologic or neuro-ophthalmologic findings before the systemic manifestations of sarcoidosis are seen. Ocular symptoms include uveitis and keratitis sicca. Musculoskeletal symptoms include myositis, bony lesions, and polyarthritis. Head, neck, and upper respiratory tract symptoms include dry cough, tonsillitis and epiglottitis; cardiac symptoms include cor pulmonale caused by pulmonary fibrosis, complete heart block, ventricular tachycardia most common arrhythmia, myocarditis, pericarditis, and congestive heart failure [9].

The mainstay of treatment is based on corticosteroids with good success rates [10]. In this case we can discuss whether nephrolithiasis was already in the context of a possible sarcoidosis because it is a common presentation of this multisystemic disease. It was essential to exclude possible infectious causes including tuberculosis in order to start treatment with corticosteroids. On further observation after 3 weeks of steroid therapy she presented with no anemia or leucopenia, and her renal function had normalized.

## Conclusion

Although sarcoidosis usually presents with pulmonary and lymph nodes involvement, in the emergency of a young adult woman with acute renal failure it becomes imperative to focus on the correct diagnosis because early therapy is essential to avoid progression to terminal renal failure .

## Consent

Written informed consent was obtained from the patient for publication of this case report and any accompanying images. A copy of the written consent is available for review by the Editor-in-Chief of this journal.

## Competing interests

The authors declare that they have no competing interests.

## Authors’ contributions

JR, MC, MF, AP, MFS and MTC dealt directly with the patient, and contributed to the writing and editing of the manuscript. RV and SS contributed to the histological examination of the kidney. All authors read and approved the final manuscript.
